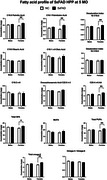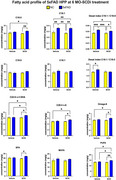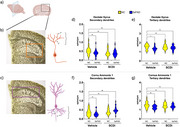# Stearoyl‐CoA desaturase inhibition leads to fatty acids normalization and improves dendritic spine density in the hippocampus of 5xFAD mouse model

**DOI:** 10.1002/alz70859_100935

**Published:** 2025-12-25

**Authors:** Marta Turri, Myriam Aubin, Laura K Hamilton, Annick Vachon, Anne Aumont, Melanie Plourde, Karl JL Fernandes

**Affiliations:** ^1^ Université de Sherbrooke, Sherbrooke, QC Canada; ^2^ Research Center on Aging (CdRV), Sherbrooke, QC Canada; ^3^ University of Shebrooke, Sherbrooke, QC Canada; ^4^ Centre de recherche sur le vieillissement, Sherbrooke, QC Canada; ^5^ Universite de Montreal/CRCHUM, Montreal, QC Canada; ^6^ University of Sherbrooke, Sherbrooke, QC Canada; ^7^ Research Center on Aging CIUSSS de L’Estrie ‐ CHUS, Sherbrooke, QC Canada

## Abstract

**Background:**

Alterations in brain lipids are a central feature of Alzheimer’s disease (AD), nevertheless therapeutic strategies targeting brain lipid metabolism are still lacking. Our lab recently reported that a pharmacological inhibitor of the fatty acid enzyme, stearoyl‐CoA desaturase (SCD), led to recovery of hippocampal synapses with associated improvements in learning and memory in the slow‐progressing 3xTg‐AD mouse model. Here, we used the 5xFAD rapidly progressing AD model to further delve into lipid metabolism disruptions in AD, and into the effect of the SCD inhibitor (SCDi) on fatty acid (FA) alterations and synapse loss.

**Method:**

Hippocampi from 5xFAD and non‐carrier control mice were collected at 5 and 8 months old (MO) for FA profiling by gas chromatography–flame ion detection (GC–FID) and for IHC for b‐amyloid, GFAP (astrocytes) and Iba‐1 (microglia). SCDi or vehicle was infused via intracerebroventricular osmotic pumps for 28 days in 5 MO 5xFAD and NC mice, and their hippocampi were processed for GC‐FID and Golgi staining for dendritic spine quantification.

**Result:**

FA alterations were apparent in female hippocampus at 5 MO (together with plaque pathology and gliosis) and worsened by the age at 8 MO, while males first showed FA alterations at 8MO (Figure 1). The C16:1/C16:0 desaturation index, parameter associated to SCD enzymatic activity, showed a significant increase in 5xFAD mice at 8 MO, but starting at 5MO in females. Treating 5xFAD females’ mice with SCDi improved dendritic spine density and normalized FA levels (Figures 2 and 3).

**Conclusion:**

These data demonstrate that SCD inhibitor treatment in a second AD mouse model, the more aggressive 5xFAD model, has beneficial effects on FA alterations and hippocampal dendritic spines. These findings add to accumulating data supporting SCD inhibition as a promising novel therapeutic target for AD.